# Metformin use and the risk of incident osteoarthritis among individuals with diabetes: a register-based nested case-control study

**DOI:** 10.1136/bmjopen-2025-115587

**Published:** 2026-04-16

**Authors:** Andrea Dell’Isola, Karin Magnusson, Aleksandra Turkiewicz, Filippo Recenti, Stefan Lohmander, Martin Englund, Ali Kiadaliri

**Affiliations:** 1 Clinical Epidemiology Unit, Orthopedics, Department of Clinical Sciences Lund, Lund University, Lund, Sweden; 2 Cluster for Health Services Research, Oslo, Norway; 3 Department of Neurosciences, Rehabilitation, Ophthalmology, Genetics, Maternal and Child Health, University of Genoa, Campus of Savona, Savona, Italy; 4 Clinical Epidemiology Research and Training Unit, Boston University School of Medicine, Boston, Massachusetts, USA

**Keywords:** EPIDEMIOLOGY, RHEUMATOLOGY, Orthopedics

## Abstract

**Abstract:**

**Objective:**

To evaluate the association between metformin use and incident osteoarthritis (OA) in people with diabetes and the impact of dosing.

**Design:**

Nested case-control study within a cohort of >1.4 million individuals from Sweden.

**Participants:**

Participants were aged 35–80 years in 2005, without diabetes or OA. We identified persons with incident diabetes between 2006 and 2016 and excluded those with OA before the diabetes diagnosis and those with an incident OA diagnosis within 3 years of the diabetes diagnosis. Cases were defined as individuals with incident OA before 2020 and were matched with up to four controls without OA in the same period, by sex, diabetes duration, birth year (±1 year) and date of diabetes diagnosis (±180 days) using incidence density sampling. Metformin use before the index date (OA diagnosis) was the main exposure. Secondary exposures were quartiles of total metformin use (defined daily doses (DDD)) and duration-adjusted use (DDD/day), reflecting average daily use. We estimated risk ratios with 95% CIs using conditional logistic regressions, adjusted for age at diabetes diagnosis, education, immigration status and comorbidities.

**Primary outcome:**

Incident OA diagnosis in primary or specialist care (International Classification of Diseases codes M15–M19).

**Results:**

We identified 4007 cases and 14 111 controls. Any metformin use was not associated with OA risk (risk ratio (RR) 1.02, 95% CI 0.93 to 1.12). Results for higher total use (0.98 (95% CI 0.86 to 1.11)) and duration-adjusted use (0.92 (95% CI 0.79 to 1.07)) showed no or inconclusive associations.

**Conclusions:**

In individuals with incident diabetes and no prior OA, metformin was not linked to a lower risk of developing OA.

**STRENGTHS AND LIMITATIONS OF THIS STUDY**

- We implemented an extended look-back period to ensure that diabetes and its medication, as well as osteoarthritis (OA), were incident, an advantage over previous studies.
- We identified 4007 cases and 14 111 controls who were free of both OA and diabetes and estimated the risk of incident OA up to 14 years after starting metformin.
- Studying metformin using electronic records of medication dispensation may not accurately represent actual usage, as overall adherence to the medication is typically low.
- Since this is an observational study, residual confounding may also be present.

## Introduction

 Osteoarthritis (OA) is one of the most prevalent and fastest-growing causes of disability worldwide.^1^ Attempts to identify disease-modifying drugs have been negative. With no approved disease-modifying treatment, repurposing medications with potential OA disease-modifying properties represents a safe alternative that could limit the costs and hurdles of novel drug development.^2^

Metformin is a first-line treatment for type 2 diabetes, and in addition to its direct role in glucose homeostasis, it exerts anti-inflammatory and immune modulatory effects.^3 4^ Due to its safety profile and low cost, metformin has been suggested as a promising drug to be repurposed for the treatment of established OA.^2^ Preclinical studies in mice and primates have suggested a reduction in cartilage degradation and synovitis with metformin treatment.^5^ A recent placebo randomised controlled trial in overweight and obese individuals with OA has suggested a potentially clinically relevant pain reduction following a 6-month metformin therapy, while a twin-design observational study suggests metformin to be able to reduce the risk of future OA diagnoses.^6^ Based on this evidence, we hypothesise that metformin may serve as a preventive treatment for clinically diagnosed OA. However, robust population-level evidence on metformin for OA prevention remains sparse.^7 8^ In this study, we address these gaps using a nested case-control study within a regional register-based incidence cohort of >1.4 million people to evaluate the association between metformin use and incident OA among individuals with diabetes (the main indication for metformin) and to estimate the impact of dose.

## Methods

### Study design and source population

**Figure 1:**
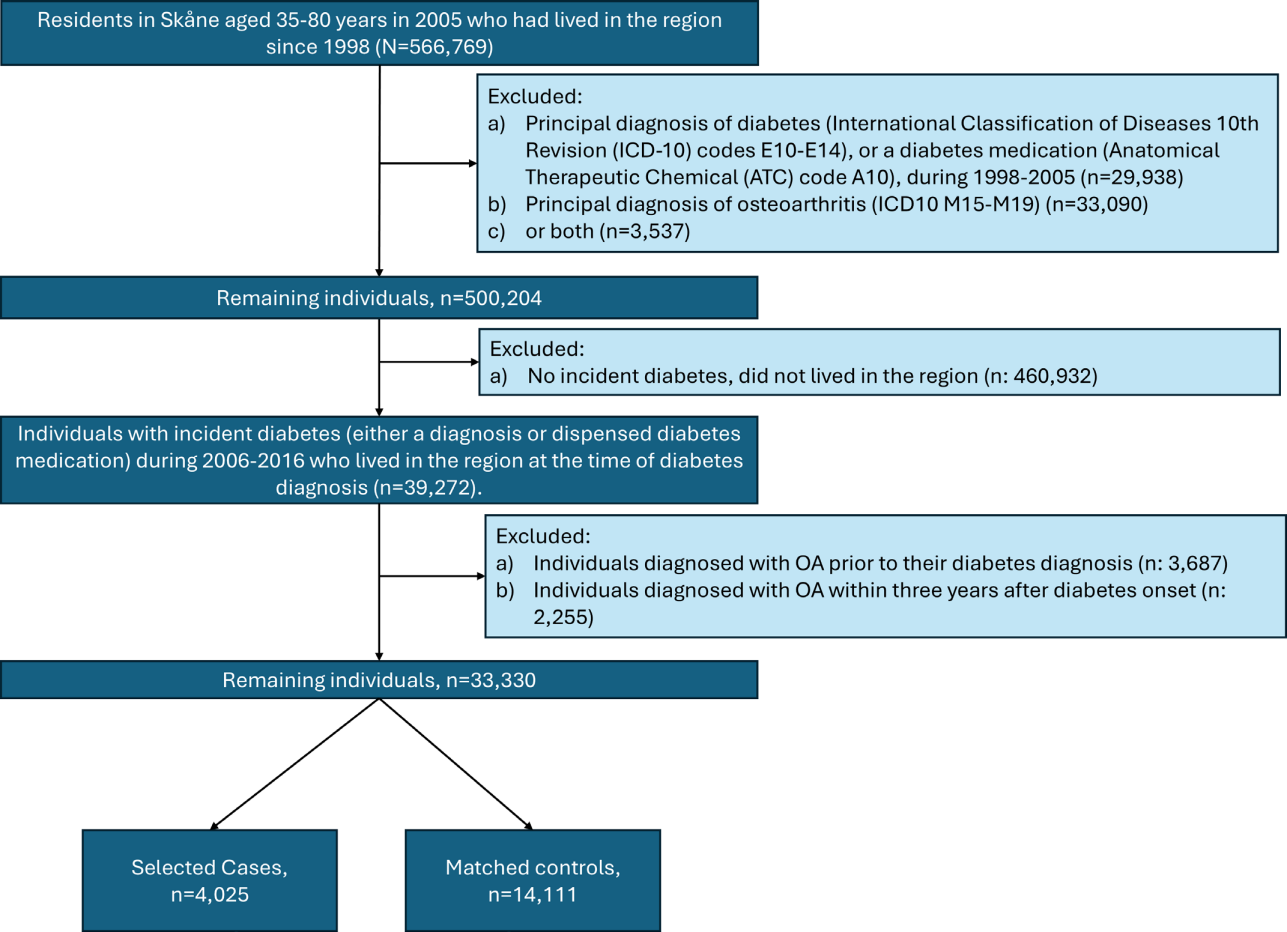
Study flowchart. OA, osteoarthritis. N,numberof individuals from source population. n, number of individuals in the study sample

This was a population-based, nested case-control study. We used four registers (the Swedish Population Register, the Longitudinal Integration Database for Health Insurance and Labour Market Studies, the Skåne Healthcare Register and the Swedish Prescribed Drug Register) comprising the entire population of Skåne, the southernmost region in Sweden, with approximately 1.4 million inhabitants (13% of the total Swedish population as of December 2020).^9^ Data from the four registers were linked using patients’ pseudo-anonymised unique personal identification number, which is assigned to all residents in Sweden by the Swedish Tax Agency. We reported the study in accordance with the REporting of studies Conducted using Observational Routinely-collected health Data (RECORD) guidelines (online supplemental file 1).^10^ Our source population included all individuals aged 35–80 years who were residents of the Skåne region in 2005 and had lived in the region since 1998 (n=566 769). We then excluded all individuals with a prevalent diabetes diagnosis (International Statistical Classification of Diseases, 10th revision (ICD-10) codes E10–E14), a diabetes medication dispensation (Anatomical Therapeutic Chemical (ATC) code A10) or OA (ICD-10 codes M15–M19). We then selected individuals with diabetes onset (diagnosis or medication) during 2006–2016 and no OA diagnosis preceding the diabetes onset or within 3 years of the diabetes onset (n=33 330). The study participant selection process is summarised in figure 1.

### Case and control definition

Among the 33 330 selected individuals, we defined cases as those with a principal OA diagnosis (diagnosis set as the main reason for the consultation) at least 3 years after diabetes diagnosis (n=4025, to avoid capturing prevalent undiagnosed OA cases and reduce protopathic bias, as the diagnostic process for diabetes often coincides with a rise in comorbid diagnoses) and randomly matched them to up to 4 controls (ie, included individuals without an OA diagnosis) by sex, diabetes duration, birth year (±1 year) and the date of diabetes diagnosis (±180 days) using incidence density sampling (ie, from those still at risk of OA at the time of the case). This implies that a control may later become a case, and the same person can be selected as a control more than once. Also, this implies that each control was at a similar stage of the diabetes disease as the matched case, reducing the risk of bias by indication, that is, where the occurrence of metformin treatment and OA could be influenced by the severity of diabetes. We used the date of diabetes diagnosis as the start of the follow-up period. This methodology allows for the estimation of risk ratios (RR) that are adjusted for all matching factors.

### Exposure

Any dispensation of metformin (Anatomical Therapeutic Chemical (ATC) codes: A10BA02, A10BD02, A10BD03, A10BD05, A10BD07, A10BD08 and A10BD10) prior to the date of incident OA diagnosis (hereafter called the index date) was used as the exposure for the main analysis. In the secondary analyses, we used exposure quartiles of the total metformin use (computed as the sum of all defined daily doses (DDD) between the first metformin initiation and the index date) and duration-adjusted total use (total daily doses divided by days since first metformin initiation to index date (DDD/day), representing average daily use), with individuals not using metformin treated as the reference group.

### Outcome

Incident OA diagnosis, defined as a recorded ICD-10 code M15–M19 in primary or specialist care, was used as the outcome.

### Statistical analysis

We performed conditional logistic regressions adjusted for age at diabetes diagnosis, education, immigration status and Elixhauser comorbidity index (1998–2005, using ICD-10 codes from primary and specialist care) to obtain risk ratios (RR) and associated 95% CIs. We had no available data on body weight. However, we did not consider this to be a significant source of confounding as both cases and controls have diabetes and may be expected to have a similar average body weight.

In the main analysis, we estimated the risk of developing OA in individuals with incident diabetes and incident use of metformin (i.e. any use) compared with individuals with incident diabetes but no metformin use. In secondary analyses, we investigated the risk associated with different levels (quartiles) of total metformin use and duration-adjusted use, with individuals not using metformin treated as the reference group. Individuals with missing values (n=212) were excluded from analyses.

We performed several sensitivity analyses to test the robustness of the results: (1) we excluded individuals with metformin dispensed only during the lag period (ie, within 3 years from the diabetes diagnosis date, n=1288); (2) we included only individuals with a diagnosis of OA between 2016 and 2019 so that all included individuals had at least 10 years between their diabetes diagnosis and OA incidence, allowing for a longer period of metformin use (n=10 569); (3) we combined the selection criteria used in analyses 1 and 2 (n=9919) and (4) we excluded individuals with the dispensation of a diabetes medication without a main diagnosis of diabetes (807 excluded, final sample 16 816) (online supplemental file 2).

### Patient and public involvement

There was no patient or public involvement in this study.

## Results

We identified 18 cases with no controls, 44 with one control, 111 with two controls, 1563 with three controls and 2289 with four controls. Baseline characteristics of cases and controls are reported in table 1.

**Table 1:** Baseline characteristics

	Cases with OA	Controls without OA
N	4007	14 111
Female, n (%)	1955 (48.8)	6859 (48.6)
Age at diabetes diagnosis, mean (SD)	62.9 (9.9)	62.7 (9.5)
Diabetes duration at index date, mean (SD)	6.4 (2.5)	6.5 (2.5)
Education, n (%)		
0–9 years	1416 (35.3)	4997 (35.4)
10–12 years	1802 (45.0)	6199 (43.9)
13+ years	764 (19.1)	2728 (19.3)
Missing	25 (0.6)	187 (1.3)
Born in Sweden, n (%)	3109 (77.6)	10 539 (74.7)
Elixhauser comorbidity index (1998–2005), n (%)		
0	2053 (51.2)	7622 (54.0)
1	1107 (27.6)	3717 (26.3)
2	539 (13.5)	1746 (12.4)
3+	308 (7.7)	1026 (7.3)

N/n, number; OA, osteoarthritis.

Among cases and controls, 3201 (79.9%) and 11 330 (80.3%) individuals, respectively, had a metformin dispensation before the index date (date of OA diagnosis) and were treated as exposed. Cases had a mean of 926.3 DDD per person (median (Q1 to Q3); 650 (75 to 1400)), while controls had 980.0 DDD per person (median 700 (75 to 1480)). Any use of metformin was not associated with a reduction of the risk of incident OA, even after adjustment for confounders (fully adjusted RR 1.02 (95% CI 0.93 to 1.12)) (table 2, online supplemental file 3). Higher total use of metformin was not associated with the risk of developing OA (Q4 (highest quartile); fully adjusted RR 0.98 (95% CI 0.86 to 1.11)). Duration-adjusted use was associated with a progressive reduction of the risk of developing OA; however, the 95% CIs did not rule out the possibility of no association (Q4 (highest quartile) fully adjusted RR 0.92 (95% CI 0.79 to 1.07)). The sensitivity analyses yielded similar results, showing no overall association between metformin use and incident OA (online supplemental files 4–7).

**Table 2:** Association of metformin use and risk of incident OA

	RR (95% CI, adjusted for matching factors*)	Adjusted RR† (95% CI)
Any use (no-use: ref)	1.01 (0.92 to 1.10)	1.02 (0.93 to 1.12)
Total use (DDD)		
No use (ref)	–	–
Q1 (lowest quartile)	1.03 (0.93 to 1.16)	1.04 (0.93 to 1.17)
Q2	1.06 (0.95 to 1.19)	1.06 (0.95 to 1.16)
Q3	0.97 (0.87 to 1.09)	0.99 (0.88 to 1.11)
Q4 (highest quartile)	0.96 (0.84 to 1.08)	0.98 (0.86 to 1.11)
Total duration-adjusted use‡ (DDD/day)		
No use (ref)	–	–
0–0.49	1.02 (0.93 to 1.13)	1.04 (0.94 to 1.14)
0.5–0.99	1.01 (0.91 to 1.12)	1.03 (0.93 to 1.14)
≥1	0.90 (0.77 to 1.04)	0.92 (0.79 to 1.07)

DDD for Q1: 7–425; Q2: 426–935; Q3: 936–1700; Q4: 1701–6490.

* Sex, diabetes duration, birth year (±1 year) and the date of diabetes diagnosis (±180 days).

† Adjusted for matching factors, age at diabetes diagnosis, education, immigration status and Elixhauser comorbidity index (1998–2005).

‡ Duration calculated starting from the first metformin dispensation.

DDD, defined daily doses; OA, osteoarthritis; ref, reference category; RR, risk ratio.

## Discussion

In a large population-based cohort of individuals with incident diabetes, starting treatment with metformin and the total dose of metformin were not associated with the risk of developing OA within 3 years and up to 14 years after starting metformin. Higher average daily doses showed inconclusive results.

Three previous studies have investigated the association between metformin and incident OA among individuals with diabetes, showing contrasting results.^7 8^ One of these studies used a prevalent new user design to compare metformin with sulfonylurea and reported that individuals receiving metformin had a 24% lower hazard of developing OA (HR, 0.76 (95% CI 0.68 to 0.85)).^7^ Another study used electronic health records to compare individuals with diabetes who received metformin with those who did not and found no association with OA incidence.^8^ Finally, a twin design study showed that individuals treated with metformin had a 10-year incidence of OA diagnosed in specialist care that was 3% lower than their twin without diabetes and not treated with metformin.^11^ In light of our findings and previous results, starting metformin medication does not appear to have a clear preventive effect on OA in individuals with diabetes.

However, evidence from a randomised controlled trial and observational studies suggests that metformin may have a clearer beneficial effect in individuals with established diseases, where it appears to reduce pain and protect against progression to total joint replacement.^6 12^ In our secondary analyses, studying metformin doses, we could not rule out a potential reduction in the risk of incident OA at higher average daily use (DDD/day). It is thus possible that certain doses used to treat diabetes may not be high enough to influence OA development and progression. Moreover, metformin therapy is characterised by overall low adherence, which can impact its effectiveness.^13 14^ Suboptimal adherence and an uncertain dose-effect relationship are likely two factors behind the contrasting results between our and previous studies.

Overall, the strength of the present study lies in the large cohort, in which we included an extensive look-back period to ensure that diabetes and its medication, as well as OA, were incident—an advantage over previous studies. Nonetheless, there are limitations to be acknowledged. We lacked data on body weight and composition, which could lead to bias if associated with diabetes therapy selections. We could not account for other diabetes medications, which might have biased the results if these drugs delayed OA onset and were more common in one exposure group. However, Glucagon-like peptide-1 (GLP-1) antagonist—the medication with the potential largest impact on OA in our population—was approved in Europe for diabetes in 2018 and for weight loss in 2022. Considering that our data cover OA only up to 2019, its impact is likely minimal. Sodium/glucose cotransporter 2 inhibitors (SGLT2i), another class of antidiabetic drugs, have been suggested to reduce OA incidence.^15^ Not accounting for SGLT2i use may also introduce bias if its use differs between cases and controls. Studying total use and duration-adjusted use of metformin may not accurately reflect the cumulative effect of a medication known to have overall low adherence. Relying on diagnosis and electronic health records to establish OA status is also a limitation, despite a previous study reporting overall high validity.^16^ Finally, the observational nature of the study limits our ability to ascertain causality.

## Conclusions

Among individuals with incident diabetes and free of OA, starting metformin was not associated with a reduction in the risk of developing OA. However, our study did not exclude that higher metformin doses could lead to a reduction in the risk of developing OA.

## Supplementary material

10.1136/bmjopen-2025-115587 online supplemental file 1

## Data Availability

Data is publicaly available upon request.
