# Supplementary material for: Metformin use and the risk of incident osteoarthritis among individuals with diabetes: a register-based nested case-control study
Source: BMJ Open. 2026 Apr 16;16(4):e115587. doi: 10.1136/bmjopen-2025-115587 (PMC13110609; doi:10.1136/bmjopen-2025-115587)
Supplement: Supplementary file 2 [file bmjopen-2025-115587.reviewer_comments.pdf]

PEER REVIEW HISTORY

BMJ Open publishes all reviews undertaken for accepted manuscripts. Reviewers are asked to complete a checklist review form and are provided with free text boxes to elaborate on their assessment. These free text comments are reproduced below.

This paper was submitted to another journal from BMJ but declined for publication following peer review. The authors addressed the reviewers' comments and submitted the revised paper to BMJ Open. The paper was subsequently accepted for publication at BMJ Open.

ARTICLE DETAILS

Title (Provisional)

Metformin use and risk of incident osteoarthritis among individuals with diabetes: a register-based nested case-control study

Authors

Dell'Isola, Andrea; Magnusson, K.; Turkiewicz, Aleksandra; Recenti, Filippo; Lohmander, Stefan; Englund, Martin; Kiadaliri, Ali

---

VERSION 1 - REVIEW

**Reviewer**	**1**
**Name**	**Reviewer 1**
**Affiliation**	
**Date**	**06-Nov-2025**
**COI**	**no**

Comments not published pending reviewer's consent

**Reviewer**	**2**
**Name**	**Reviewer 1**
**Affiliation**	
**Date**	**06-Nov-2025**
**COI**	**no**

Comments not published pending reviewer's consent

**Reviewer**	**3**
**Name**	**Døssing, Anna**
**Affiliation**	
**Date**	**06-Nov-2025**
**COI**	**no**

This manuscript uses a nested case-control study to investigate whether metformin use in people with diabetes is associated with the development of osteoarthritis. In people with incident diabetes without prior OA, metformin use was not associated with a reduced risk of OA development. The manuscript is well written, easy to follow, and has good coherence be-tween the aim, results, and conclusion. This study is interesting and appears well-executed. I don't have the methodological background to evaluate the methods used in this study properly.

Minor comments and points to consider in the discussion are added below

INTRODUCTION
1. It is not clear to me why you choose to focus on people with diabetes. Can you maybe add a sentence or two in the background to clarify this?
2. Did you have a hypothesis before you started the study? If so, please disclose this.

METHODS
3. Tabl 1: Elixhauser comorbidity index appears – please describe how this data is collected in the method section – ICD10 data?

RESULTS AND DISCUSSION
It may be worthwhile to examine the results of the current study in the context of the RCT referenced in 6.
4. Is it possible to adjust for weight or do a subgroup analysis of obese people?
5. Does the site of osteoarthritis matter? Would you expect results to differ, e.g., among people with knee OA and hand OA?

Antidiabetic drugs are of great interest when it comes to repurposing drugs. They may have different impacts on OA (PMID: 39833406).
6. Is it possible to consider how different antidiabetic drugs may impact the results?
7. Has the introduction of GLP1 receptor agonists influenced the results of the current study?

VERSION 1 - AUTHOR RESPONSE

Reviewer: 3

Reviewer comment:
This manuscript uses a nested case-control study to investigate whether metformin use in people with diabetes is associated with the development of osteoarthritis. In people with incident diabetes without prior OA, metformin use was not associated with a reduced risk of OA development. The manuscript is well written, easy to follow, and has good coherence between the aim, results, and conclusion. This study is interesting and appears well-executed. I don't have the methodological background to evaluate the methods used in this study properly.
Minor comments and points to consider in the discussion are added below.

INTRODUCTION

1. It is not clear to me why you chose to focus on people with diabetes. Can you maybe add a sentence or two in the background to clarify this?

Response: We thank the expert reviewer for the comment. We agree that this should be specified to aid understanding of the approach. The reason is that metformin is rarely used for treating other conditions. There are some indications for its use to treat polycystic ovary syndrome (PCOS), but this is a rare condition. Thus, to study metformin use with observational data, we were forced to limit the population to those with a common indication for metformin to minimise bias by indication.

Changes to the manuscript: added a sentence in the introduction.

Line 97: In this study, we address these gaps using a nested case–control study within a regional register-based incidence cohort of >1.4 million people to evaluate the association between metformin use and incident OA among individuals with diabetes (the main indication for metformin), and to estimate the impact of dose.

Reviewer comment:

2. Did you have a hypothesis before you started the study? If so, please disclose this.
Response: We thank the reviewer for the comment. We have added the hypothesis in the introduction

Changes to the manuscript: added a sentence in the introduction.

Line 92: Based on this evidence, we hypothesise that metformin may serve as a preventive treatment for clinically diagnosed OA.

METHODS

Reviewer comment:

3. Table 1: Elixhauser comorbidity index appears – please describe how this data is collected in the method section – ICD10 data?
Response: We thank the reviewer for noticing this.

Changes to the manuscript: we have specified that we used ICD-10 codes from primary and specialist care

Line 137: We performed conditional logistic regressions adjusted for age at diabetes diagnosis, education, immigration status, and Elixhauser Comorbidity index (1998-2005, through ICD-10 codes from primary and specialist care) to obtain RR and associated 95% confidence intervals (95% CIs).

RESULTS AND DISCUSSION

Reviewer comment:

It may be worthwhile to examine the results of the current study in the context of the RCT referenced in 6.

Response: We agree with the reviewer that this can be important.

Changes to the manuscript: we have added a brief sentence discussing the results from the mentioned trial while also including other studies investigating the association between metformin and progression to joint replacement.

Line 185: However, evidence from an RCT and observational studies suggests that metformin may have a more clear beneficial effect in individuals with established diseases, where it appears to reduce pain, and protect against progression to total joint replacement[6, 12].

Reviewer comment:
4. Is it possible to adjust for weight or do a subgroup analysis of obese people?

Response: We thank the reviewer for the comment. Unfortunately, we have no data on body weight or body composition. We acknowledged this as a limitation.

Changes to the manuscript: no changes were made

Reviewer comment:
5. Does the site of osteoarthritis matter? Would you expect results to differ, e.g., among people with knee OA and hand OA? Antidiabetic drugs are of great interest when it comes to repurposing drugs. They may have different impacts on OA (PMID: 39833406).

Response: We agree with the author that this is a very interesting question. We are unfortunately unable to provide estimates by joint due to the low number of hand OA cases. This is also due to the fact that in a nested-case-control design, changing the outcome (i.e. the joint affected by OA) would require a separate matching.

Changes to the manuscript: we added a sentence

Reviewer comment:
6. Is it possible to consider how different antidiabetic drugs may impact the results?

Response: We thank the reviewer for the comment. We agree that this is a limitation.

Changes to the manuscript: We have added a statement in the limitations section to acknowledge the potential presence of bias due to other medications. We have also included a statement about GLP-1 antagonist to respond to comment n.7 from the reviewer.

Line 199: We could not account for other diabetes medications, which might have biased the results if these drugs delayed OA onset and were more common in one exposure group. However, GLP-1—the medication with the potential largest impact on OA in our population—was approved in Europe for diabetes in 2018 and for weight loss in 2022. Considering that our data cover OA only to 2019, its impact is likely minimal.

Reviewer comment:
7. Has the introduction of GLP1 receptor agonists influenced the results of the current study?

Response: We thank the reviewer for the comment. Considering that our data extend only to 2019 for OA and 2016 for the diagnosis of diabetes (due to the 3-year lag-time imposed) and that approval in Europe of GLP1 for diabetes happened in 2018, and for weight loss in 2022, we believe that its impact on the estimate is minimal. However, as for the comment above, we recognised that not being able to account for other medications is a limitation.

Changes to the manuscript: We have also included a statement about GLP-1 antagonist in the limitations section:

Line 199: We could not account for other diabetes medications, which might have biased the results if these drugs delayed OA onset and were more common in one exposure group. However, GLP-1—the medication with the potential largest impact on OA in our population—was approved in Europe for diabetes in 2018 and for weight loss in 2022. Considering that our data cover OA only to 2019, its impact is likely minimal.

VERSION 2 - REVIEW

**Reviewer**	**3**
**Name**	**Døssing, Anna**
**Affiliation**	**The Parker Institute, Rheumatology**
**Date**	**22-Jan-2026**
**COI**	**None**

The conflicting results regarding metformin deserve attention, and a case-control study like this is a nice supplement to existing knowledge. This study is interesting and appears well-executed. The main limitations are the lack of data on body weight and the failure to account for other anti-diabetic drugs (GLP1 and SGLT2). Both limitations are mentioned in the manu-script. I have already peer-reviewed this manuscript (reviewer 3), and I am happy to see the authors' adjustments and reply. Some minor questions that remain are

RESULTS/DISCUSSION

1. I understand the low number of hand OA cases does not allow for analysis of this, and that you do not have access to the weight data. If possible, it would still be interesting to see results from weight-bearing joints compared to non-weight-bearing joints as a proxy for the impact of weight and OA site.

2. I appreciate the inclusion of GLP1 in the discussion. Given the debate about SLGT2 this might deserve mention as well.

**Reviewer**	**4**
**Name**	**Dack, Kyle**
**Affiliation**	**University of Bristol, MRC Integrative Epidemiology Unit**
**Date**	**21-Jan-2026**
**COI**	**None**

Statistical review summary:

The statistical methodology follows best practices and I do not think there are any notable risks or sources of bias not already acknowledged. The paper is written clearly, with clear reporting and reasonable interpretation of the data. However, the reporting in the main text is somewhat minimal, and would benefit from more information in the methods and results sections (e.g. variable definitions, inclusion criteria, sensitivity analyses).

Specific comments:

1. Prior peer review: I note that the submission contains responses from what looks to be a prior peer-review process, either with BMJ Open or elsewhere I am unsure. I found all the responses to be reasonable. Particularly regarding the statistics, I agree with the authors that there is not risk of confounding from body weight, because both cases and controls were restricted to those with diabetes. At most, it may add uncertainty to the estimates, but this is more or less already acknowledged in the limitations section. Given that two prior reviewers picked up on this (indicating that readers are likely to also query it), perhaps a justification could also be added to the methods when discussing confounders?

Abstract:

2. No suggestions.

Introduction:

3. Clear and aligned with STROBE/RECORD – no suggestions.

Methods:

4. Inclusion / exclusion criteria are reported in Figure 1 but not in the main methods text. While this is not a requirement of STROBE or RECORD, it is common and convenient for readers. If there is spare word count I recommend considering to add this. At minimum I think line 119 should make it clearer that the figure is where the exclusion criteria can be found.

5. Line 134: The exposures are described in details, but the outcome was not made explicit, nor any accompanying covariates. This relates to STROBE item 7/8 regarding variables and data sources, and RECORD item 7.1, so I suggest adding a brief section to define these items.

6. Line 145: education was adjusted for, but it looks like there was missing data for this variable only. How was this handled – were those without education excluded, or imputation used?

7. Line 152: It would be helpful to give a broad overview of sensitivity analyses and their purpose in the main text. Also in the results (line 165) this seems very brief and under-utilised. I don't think space should be an issue given that generally the methods and results are relatively short.

8. Line 152: I think "results" is missing a line break, as in my pdf copy this heading was not on a new line.

Results:

9. Line 153: in total this section reports 4025, which aligns with the prior reported case count (line 123). However, the analysis beginning on line 156 includes only 3201 cases, meaning 824 cases were dropped. I imagine this includes 18 with no control, but which others? Adding details either here and/or in the methods regarding who was included in the main analysis would make this more transparent.

10. Table 2. I suggest in the table or footnotes defining the quartile ranges, which will make this more interpretable and enable comparison with any future studies.

11. Table 2. The categories of total duration adjusted use overlap – two contain “0.5”.

VERSION 2 - AUTHOR RESPONSE

Reviewer: 3

Miss Anna Døssing, The Parker Institute

Reviewer's comment:
The conflicting results regarding metformin deserve attention, and a case-control study like this is a nice supplement to existing knowledge. This study is interesting and appears well-executed. The main limitations are the lack of data on body weight and the failure to account for other anti-diabetic drugs (GLP1 and SGLT2). Both limitations are mentioned in the manu-script. I have already peer-reviewed this manuscript (reviewer 3), and I am happy to see the authors' adjustments and reply. Some minor questions that remain are

Reviewer's comment:
1. I understand the low number of hand OA cases does not allow for analysis of this, and that you do not have access to the weight data. If possible, it would still be interesting to see results from weight-bearing joints compared to non-weight-bearing joints as a proxy for the impact of weight and OA site.

Response: We agree that this would be interesting; however, as we have noted, there are very few cases involving non-weight-bearing joints. Conducting joint-specific analyses in a matched case-control design would require extensive re-analyses, including repeating the matching process. Additionally, our original hypothesis was that metformin should have an effect on any joint. Therefore, we prefer not to carry out further analyses at this stage.

Reviewer's comment:
2. I appreciate the inclusion of GLP1 in the discussion. Given the debate about SLGT2 this might deserve mention as well.

Response: We thank the reviewer for the suggestion.

Changes to the manuscript: We have now added a sentence mentioning potential confounding effect from SGLT2i.

Line 210: SGLT2 inhibitors (SGLT2i), another class of antidiabetic drugs, have been suggested to reduce OA incidence[15]. Not accounting for SGLT2i use may also introduce bias if its use differs between cases and controls.

Reviewer: 4
Dr. Kyle Dack, University of Bristol
Comments to the Author:
Statistical review summary:
The statistical methodology follows best practices and I do not think there are any notable risks or sources of bias not already acknowledged. The paper is written clearly, with clear reporting and reasonable interpretation of the data. However, the reporting in the main text is somewhat minimal,

and would benefit from more information in the methods and results sections (e.g. variable definitions, inclusion criteria, sensitivity analyses).

Specific comments:

Reviewer's comment:
1. Prior peer review: I note that the submission contains responses from what looks to be a prior peer-review process, either with BMJ Open or elsewhere I am unsure. I found all the responses to be reasonable. Particularly regarding the statistics, I agree with the authors that there is not risk of confounding from body weight, because both cases and controls were restricted to those with diabetes. At most, it may add uncertainty to the estimates, but this is more or less already acknowledged in the limitations section. Given that two prior reviewers picked up on this (indicating that readers are likely to also query it), perhaps a justification could also be added to the methods when discussing confounders?

Response: We agree with the reviewer that readers may be likely to wonder about the impact of the lack of BMI in the analysis. We have added a sentence in the methods as suggested.

Changes to the manuscript

Line 144: We had no available data on body weight. However, we did not consider this to be a significant source of confounding as both cases and controls have diabetes and may be expected to have a similar average body weight.

Abstract:

Reviewer's comment:
2. No suggestions.

Introduction:

Reviewer's comment:
3. Clear and aligned with STROBE/RECORD – no suggestions.

Methods:

Reviewer's comment:
4. Inclusion / exclusion criteria are reported in Figure 1 but not in the main methods text. While this is not a requirement of STROBE or RECORD, it is common and convenient for readers. If there is spare word count I recommend considering to add this. At minimum I think line 119 should make it clearer that the figure is where the exclusion criteria can be found.

Response: We agree with the reviewer. We had reported the inclusion criteria in the figure to meet the world limit for a brief report.

Changes to the manuscript: We have now added a paragraph detailing the population selection process

Line 110: Our source population included all individuals aged 35-80 who were residents of the Skåne region in 2005 and had lived in the region since 1998 (N: 566,769). We then excluded all individuals with a prevalent diabetes diagnosis (ICD-10 codes W10-E14), or a diabetes medication dispensation (Anatomical Therapeutic Chemical (ATC) code A10), or OA (ICD-10 M15-M19). We then selected individuals with diabetes onset (diagnosis or medication) during 2006-2016 and no OA diagnosis preceding the diabetes onset or within three years of the diabetes onset (n:33,330).

Reviewer's comment:
5. Line 134: The exposures are described in details, but the outcome was not made explicit, nor any accompanying covariates. This relates to STROBE item 7/8 regarding variables and data sources, and RECORD item 7.1, so I suggest adding a brief section to define these items.

Response: We agree with the reviewer that such a section is warranted

Changes to the manuscript: We have now added an outcome section:

Line 139: Incident OA diagnosis, defined as a recorded ICD10 code M15-M19 in primary or specialist care, was used as the outcome.

Reviewer's comment:
6. Line 145: education was adjusted for, but it looks like there was missing data for this variable only. How was this handled – were those without education excluded, or imputation used?

Response: We agree with the reviewer that this should be stated in the manuscript.

Changes to the manuscript: We have now added a line specifying that the 212 individuals with missing education were dropped.

Line 151: Individuals with missing values (n:212) were excluded from the analyses.

Reviewer's comment:

7. Line 152: It would be helpful to give a broad overview of sensitivity analyses and their purpose in the main text. Also in the results (line 165) this seems very brief and under-utilised. I don't think space should be an issue, given that generally the methods and results are relatively short.

Response: We agree with the reviewer that more details may improve the manuscript. However, we believe that the statement in the results should be brief, as the sensitivity analyses confirm the main finding and add very little value for the general reader other than showing that changing the criteria for selecting the sample and define exposure has not in

Changes to the manuscript: We have now detailed the sensitivity analyses in the methods and added a short sentence to the results

Line 153: We performed several sensitivity analyses to test the robustness of the results: 1) we excluded individuals with metformin dispensed only during the lag period (i.e. within 3 years from the diabetes diagnosis date, n=1,288); 2) we included only individuals with a diagnosis of OA between 2016-2019, so that all included individuals had at least 10 years between their diabetes diagnosis and OA incidence, allowing for a longer period of metformin use (n=10,569); 3) we combined the selection criteria used in analyses 1 and 2 (n=9,919); 4) We excluded individuals with the dispensation of a diabetes medication without a main diagnosis of diabetes (807 excluded, final sample 16,816)(**supplementary file 2**).

Line 176: The sensitivity analyses yielded similar results, showing no overall association between metformin use and incident OA (supplementary files

Reviewer's comment:
8. Line 152: I think "results" is missing a line break, as in my pdf copy this heading was not on a new line.
Response: We thank the reviewer for noticing this inconsistency.

Changes to the manuscript: We have now corrected the error.

Results:

Reviewer's comment:
9. Line 153: in total this section reports 4025, which aligns with the prior reported case count (line 123). However, the analysis beginning on line 156 includes only 3201 cases, meaning 824 cases were dropped. I imagine this includes 18 with no control, but which others? Adding details either here and/or in the methods regarding who was included in the main analysis would make this more transparent.

Response: We thank the reviewer for the comment. the 3201 are cases who have received metformin, and were treated as exposed.

Changes to the manuscript: We have now modified the sentence to clarify that those are the individuals exposed to metformin.

Line 168: Among cases and controls, 3,201 (79.9%) and 11,330 (80.3%) individuals, respectively, had a metformin dispensation before the index date (date of OA diagnosis) and were treated as exposed.

Reviewer's comment:
10. Table 2. I suggest in the table or footnotes defining the quartile ranges, which will make this more interpretable and enable comparison with any future studies.

Response: We thank the reviewer for the suggestion.

Changes to the manuscript: We have defined the quartile in the footnote of the table.

Reviewer's comment:
11. Table 2. The categories of total duration adjusted use overlap – two contain "0.5".

Response: We thank the reviewer for spotting this inconsistency.

Changes to the manuscript: We have corrected the table

Reviewer: 3
If you have selected 'Yes' above, please provide details of any competing interests.: Not applicable

Reviewer: 4
If you have selected 'Yes' above, please provide details of any competing interests.: NA
